# Comparative effectiveness of antihypertensive treatment for older children with primary hypertension: study protocol for a series of n-of-1 randomized trials

**DOI:** 10.1186/s13063-015-1142-y

**Published:** 2016-01-08

**Authors:** Joyce P. Samuel, Joshua A. Samuels, Lauren E. Brooks, Cynthia S. Bell, Claudia Pedroza, Donald A. Molony, Jon E. Tyson

**Affiliations:** 1 Division of Pediatric Nephrology and Hypertension, University of Texas Health Science Center at Houston (UTHealth) McGovern Medical School, 6431 Fannin, MSB 3.121, Houston, Texas 77030 USA; 2 UTHealth McGovern Medical School, Houston, Texas USA; 3 Division of Renal Diseases and Hypertension, UTHealth McGovern Medical School, Houston, Texas USA

**Keywords:** Antihypertensive medications, N-of-1 trial, Pediatrics, Hypertension, Comparative effectiveness research

## Abstract

**Background:**

Children are increasingly being diagnosed with primary hypertension. The absence of comparative effectiveness research of antihypertensive medications in children has contributed to considerable differences in prescribing practices among physicians treating children with primary hypertension. Even if parallel-group trials had established a best overall choice for most of these children, the best medication for an individual may differ from the best overall medication.

**Methods/design:**

This project consists of a series of systematically administered n-of-1 trials among older children to verify the need for ongoing antihypertensive treatment and, if so, to identify the preferred single drug therapy from among the three major classes of drugs commonly used for primary hypertension (angiotensin-converting enzyme inhibitors, calcium channel blockers, and diuretics). We will determine whether one of these is the preferred therapy for the great majority of patients. The “preferred” therapy is the drug which produces normal ambulatory blood pressure, with the greatest reduction in blood pressure without unacceptable side effects. We will recruit 50 patients from the Houston Pediatric and Adolescent Hypertension Program clinic. For each patient, the three drugs will be prescribed in random order and each drug will be taken for 2 weeks. The effectiveness of each therapy will be measured with 24-h ambulatory blood pressure monitoring, and tolerability will be assessed using a side effect questionnaire. Participants will rotate through treatment periods, repeating drugs and adjusting doses until the preferred therapy is identified. In assessing whether one of the medications is most effective for the majority of subjects, the primary outcome will be the percentage of participants for whom each drug is selected as the preferred therapy. We hypothesize that no drug will be selected for the great majority of the subjects, a finding that would support consideration of clinical use of n-of-1 trials. Secondary analyses will explore whether patient characteristics predict which medication will be selected as a preferred drug.

**Discussion:**

This study will help optimize care of participating patients and provide evidence regarding the usefulness of n-of-1 trials in identifying appropriate treatment for children with hypertension and potentially other disorders.

**Trial registration:**

Clinicaltrials.gov NCT02412761 (registered 4/8/2015).

## Background

Hypertension is one of the most important modifiable risk factors for adverse health outcomes: premature death, renal failure, stroke, and myocardial infarction are each reduced when blood pressure is controlled [1–8]. Hypertension is increasingly diagnosed in children, and effective treatment will be particularly important to reduce the ultimate years of healthy life lost to these problems.

Unfortunately, only sparse data from clinical trials inform the use of different antihypertensive agents in children [9]. As a result, the best first-line therapy for children has not been defined for national guidelines [10], and clinical practice is highly variable [11–13]. In a survey of North American pediatric nephrologists, 47 % chose angiotensin-converting enzyme (ACE) inhibitors; 37 %, calcium channel blockers, and 15 % diuretics as initial therapy in children with primary hypertension [14].

Large parallel-group randomized clinical trials (RCTs) are generally required to determine which therapy is the preferred initial therapy that on average has the greatest overall benefit. However, that therapy may be ineffective or harmful for some patients [15]. Parallel-group RCTs are often far underpowered to identify important subgroup differences or treatment interactions needed to distinguish such patients [16]. Additionally, subgroup analyses cannot unravel the complex interplay between multiple characteristics that are simultaneously at work within a given individual to modify the treatment effect [17].

In some circumstances the best treatment for individual patients may be identified in n-of-1 trials. Such trials are systematically administered, single patient RCTs in which the patient serially receives multiple therapies in a random order [18]. These trials can include allocation concealment, blinding, and all other features used to minimize bias in traditional parallel-group RCTs [19]. Unlike traditional RCTs, n-of-1 trials can facilitate treatment recommendations for each individual patient based on his or her responses to the different therapies [20]. N-of-1 trials provide a formal and more rigorous assessment than the informal “trial of therapy” often used clinically in prescribing an antihypertensive agent—an approach that can be biased due to inaccurate or infrequent assessments of blood pressure, failure to assess more than one medication, absence of predefined criteria for success, and physician reluctance to change therapy. By involving patients and their families in verifying the presence of hypertension and identifying the preferred therapy, n-of-1 trials might reduce unnecessary treatment and increase adherence when treatment is needed [21–23].

Guyatt and colleagues, who pioneered n-of-1 trials, have described the conditions for which they are most useful [24]. The condition should be chronic and require prolonged treatment; the therapies should have a rapid onset and offset of action; the response to treatment should be accurately measurable; and the physician should be willing to consider multiple therapies. Such trials have been performed in a variety of conditions, including chronic obstructive lung disease, osteoarthritis, neuropathic pain, palliative care, traumatic brain injury, and attention deficit-hyperactivity disorder [25–30]. The treatment of primary hypertension in children meets these criteria and is equally well suited for an n-of-1 trial.

## Methods

**Fig. 1:**
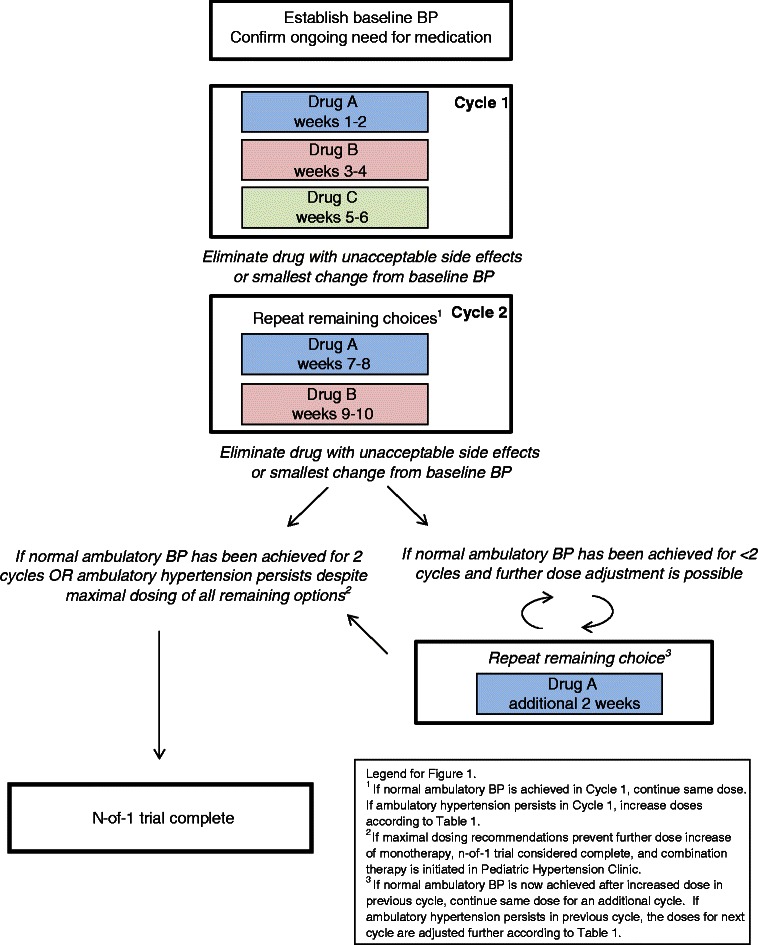
N-of-1 design schema

This is a series of n-of-1 trials to compare the effectiveness and tolerability of lisinopril, amlodipine, and hydrochlorothiazide within each patient. Each of these therapies has previously been proven to be efficacious in blinded placebo-controlled RCTs [31–33]. The preferred therapy for each patient is defined a priori as the medication which yields normal ambulatory blood pressure (BP), with the greatest reduction in ambulatory wake mean systolic BP compared to baseline BP and without the presence of unacceptable side effects. Each n-of-1 trial consists of a series of 2-week treatment periods which are repeated until the preferred therapy is identified (Fig. 1). Once the trial is complete, the patient resumes usual care in the Pediatric Hypertension Clinic.

### Study objectives and hypothesis

The primary objective is to determine whether there are individual differences in the response to different drug classes. We hypothesize that no drug will be selected for 80 % or more of the participants, a finding that might prompt consideration of n-of-1 trials in clinical practice before selecting a therapy for long-term administration. The secondary objective is to explore whether there are baseline characteristics associated with the choice of the preferred therapy for each patient.

### Study setting, eligibility criteria, and recruitment

This study is located in Houston, Texas, with recruitment occurring from patients attending the Houston Pediatric and Adolescent Hypertension Program (HPAHP) clinic at the University of Texas Health Science Center at Houston (UTHealth). Patients receiving pharmacologic antihypertensive therapy or recently referred patients who meet criteria for treatment based on ambulatory blood pressure monitoring (ABPM) are invited to participate if they are aged 9–22 years and have primary hypertension. Patients less than 9 years old are not included because younger patients have a higher likelihood of secondary hypertension, and would be less likely to comply with the repeated ABPMs required for the n-of-1 trial. All patients in this clinic undergo evaluation for secondary causes of hypertension based on national guidelines [10]. Only those patients without a compelling indication to select one particular medication (such as choosing ACE inhibitors or angiotensin receptor blockers in patients with diabetes) are considered eligible for participation. Patients with a specific contraindication for any of the three therapies are excluded.

The n-of-1 trial is offered to all eligible patients as an optional program. Patients are informed that participation is voluntary, and they can withdraw at any time. Verbal informed consent is obtained from each participant.

### Blinding and randomization

We have made no attempt to alter the medications to make them identical and indistinguishable to the patients, partly because of feasibility, cost, and concern that their effects in clinical practice might be inadvertently altered. During the n-of-1 trial, when comparing the effectiveness and tolerability of each therapy with the family, the physician is blinded to which drug was tested in each time period by using a summary sheet of ABPM results and side effects which refers to treatments as first, second, or third used. The order of the medications given is randomized each time they are compared for a patient using an online random number generator [34].

### Blood pressure definitions

Ambulatory hypertension is defined as the combination of 1) wake or sleep mean ambulatory systolic blood pressure (SBP) or diastolic blood pressure (DBP) greater than the height-sex referenced 95^th^ percentile and 2) BP load > 25 %, based on standardized values from the 2014 American Heart Association Scientific Statement on pediatric ABPM [35]. For patients aged ≥ 18 years, ambulatory hypertension is defined by adult criteria [36].

Normal ambulatory blood pressure is defined as both wake and sleep mean ambulatory SBP and DBP less than the height-sex referenced 95^th^ percentile.

### N-of-1 trial design

#### Establish baseline blood pressure

Baseline BP is defined as the wake mean SBP on the most recent 24-h ambulatory blood pressure monitoring (ABPM) off antihypertensive therapy. If it has been over a year since the last ABPM off therapy, the n-of-1 trial begins with a washout of previous antihypertensive therapy for at least 2 weeks followed by a repeat ABPM to confirm the need for ongoing therapy and to establish the baseline BP.

#### Treatment cycles

The medications are prescribed in random order at typical starting weight-based doses (Table 1). The first week of each 2-week treatment period is considered a washout period, in which the effects of the previous treatment are expected to diminish and the effects of the current treatment commence [37–39]. No assessment of BP reduction is taken during the washout period. At the end of the second week (day 13–14), ABPM is performed for a 24-h period with readings every 30 min (using SpaceLabs Ultralite 90217 monitors, SpaceLabs, Inc., Issaquah, WA, USA).

**Table 1:** Dose adjustment plan

	Initial dose	Dosing adjustment	Maximum dose
Amlodipine	0.1 mg/kg/dose Qday (max 5 mg/day)	Double dose	10 mg/day
Hydrochlorothiazide	1 mg/kg/dose Qday (max 25 mg/day)	Double dose	50 mg/day or 3 mg/kg/day
Lisinopril	0.1 mg/kg/dose Qday (max 10 mg/day)	Double dose	40 mg/day or 0.6 mg/kg/day

A clinic visit is completed on day 14 of each 2-week treatment period. At this visit, clinic BP is measured using standard methodology [10] with an oscillometric device (Spot Vital Signs LXi, Welch Allyn, Skaneateles Falls, NY, USA [40]) for four repeated seated measurements. In addition, at each visit the patient is weighed, interim medical history is obtained, side effects are assessed using a questionnaire, pill count is performed, ABPM activity diary is confirmed, and ABPM data is downloaded.

Cycle 1 is defined as the initial 6 weeks of treatment periods, during which the three options are assessed for 2 weeks each (Fig. 1). At the clinic visit marking the end of Cycle 1, the physician, patient, and parents discuss the comparative effectiveness and tolerability of each of the tested options. The drug that produces either an unacceptable side effect or the smallest decrease in ambulatory wake SBP will be removed from consideration. The remaining two drugs are repeated for 2-week treatment periods (Cycle 2) in random order to confirm which drug yields the greatest reduction in BP without unacceptable side effects. If normal ambulatory blood pressure is achieved in Cycle 1 with either of the two drugs to be repeated, the doses are not changed for Cycle 2. If ambulatory hypertension persists in Cycle 1 on all tested therapies, the doses for the medications are increased for the subsequent treatment periods according to a preplanned protocol (Table 1).

#### Trial conclusion

The trial is concluded after a drug has been shown to produce normal ambulatory BP on at least two treatment periods without unacceptable side effects. If more than one drug meets these criteria, the physician and the family employ shared decision making to determine the final preferred therapy, based on greatest average reduction in wake BP or side effect experience. If the patient or family chooses to withdraw from the n-of-1 trial before Cycle 2 can be completed, the data from Cycle 1 is used to determine the preferred drug. If none of the treatments result in normal ambulatory BP despite maximal doses, the trial is concluded and the patient returns to usual care at the Pediatric Hypertension Clinic to begin a combination therapy.

### Patient measures

#### Ambulatory BP monitoring

We use ABPM instead of clinic BP measurements to assess BP reduction on treatment for several reasons. Multiple measurements are obtained with each 24-h reading, resulting in reduced variability and greater reproducibility of measurements compared to office BP readings [41–45]. This provides greater precision in our ability to discriminate between the effects of each drug within a single patient. ABPM allows for blinded collection of BP measurements. ABPM eliminates the white-coat effect, which may vary by antihypertensive drug class [46]. ABPM has been shown to be an important tool in evaluating the efficacy of BP medications in adults [47, 48], in discriminating the comparative effectiveness of different antihypertensive drugs in adults [49, 50], and has been suggested as a superior measurement tool in hypertensive children [41, 51].

#### Side effects

**Fig. 2:**
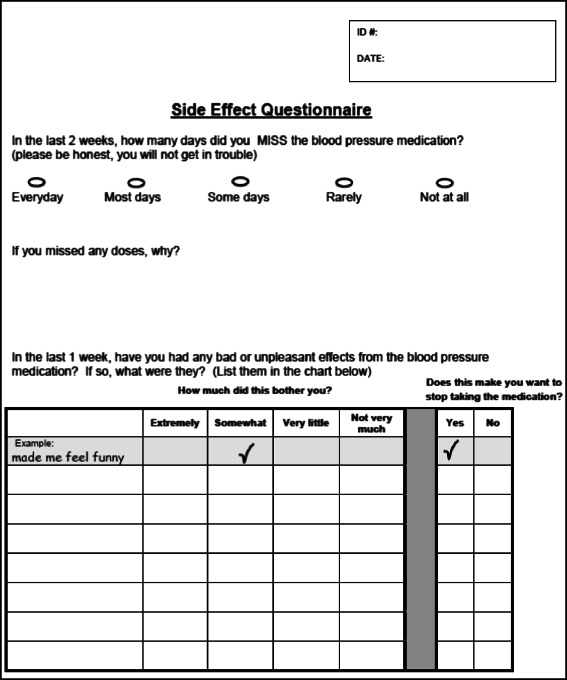
Side effect questionnaire

A side effect questionnaire has been developed for this project to identify unacceptable side effects (Fig. 2). The questionnaire includes an open-ended prompt to list all unpleasant effects from the medication, with a rating scale for how bothersome the effect is and whether it is considered unacceptable. The questionnaire was piloted in a sample group of adolescent patients in our clinic. We used the technique of translation with back-translation to create a Spanish language version of the questionnaire.

#### Safety and adverse events

If the patient takes either lisinopril or hydrochlorothiazide for the first time during the course of the n-of-1 trial, blood monitoring is performed two weeks after starting the medication as per usual clinical practice. If laboratory testing shows abnormal chemistry (excessive change in serum creatinine or potassium levels), then that treatment will be removed from consideration as a preferred drug. BP will be checked in clinic and in the ambulatory setting every two weeks. If BP on a particular treatment is considered by the physician to be dangerously high or low for that patient, the drug will not be repeated.

#### Demographic data and baseline characteristics

For all participants, at the first visit we collect information on patient age, gender, weight, height, body-mass index, race/ethnicity, self-reported exercise activity (number of hours of exercise in a usual week), and insurance coverage. We also collect parental data on age, marital status, total number of children in the household, race/ethnicity, employment, and educational attainment.

### Adherence to study procedures

We anticipate that the extra effort required to participate in the n-of-1 trial compared to usual care (frequent clinic visits and the repeated use of 24-h ABPM) will deter some families from participation. In order to maximize adherence, we utilize concepts derived from motivational interviewing, using an empathetic approach to help families explore and resolve the potential hindrances to compliance with study procedures before they begin the n-of-1 trial. We also provide external motivation with financial incentives to the patient, offering gift cards in gradually increasing amounts (ranging from $15–$40 per card) for each adequate ABPM reading to motivate the participants to comply with repeated ABPMs as they progress through the n-of-1 trial. An adequate ABPM study must contain at least 18 h of measurements.

We expect that some families might find it difficult to present to our clinic during regular office hours for visits every 2 weeks. With that in mind, for all visits associated with the n-of-1 trial, we offer extended office hours and validated parking, and we do not charge patients or insurance companies for the visits or ABPM interpretation.

Adherence to the prescribed therapy is determined using a pill count at each visit. Percent adherence is calculated as ([number of pills taken] ÷ [number of pills expected to have been taken]) × 100.

#### Acceptability questionnaire

At the final visit, participants complete a survey to assess the acceptability of the n-of-1 trial. Questions are asked on a four-point response scale, addressing whether the monetary incentives (gift cards and parking validations) influenced their willingness to participate. Open-ended questions explore the importance of after-hour options and the most satisfying and most difficult aspects of the n-of-1 trial.

### Power calculation and analytic plan

The aggregate data from all completed n-of-1 trials will be combined to assess the primary outcome, which is the percentage of patients for whom each drug is selected as the preferred therapy. For our primary aim of determining if any drug will be selected for 80 % or more of the subjects, a frequentist approach will be used to calculate for each drug a point estimate and 95 % binomial confidence interval for the proportion of individuals who prefer that drug. Our primary hypothesis of treatment equipoise will be confirmed if the upper limit of the 95 % confidence interval does not reach 80 % for any of the drugs (that is, the observed proportion of individuals who prefer any one drug will be less than 80 %).

With the expectation of recruitment of 75 subjects (2 to 3 per month) and 33 % drop-out rate, we expect to conduct 50 separate n-of-1 trials over the course of the funding period. With this number of patients our power (at an alpha error of 0.05) to reject our hypothesis is estimated to be ≥ 80 % if in truth any drug is preferred by 60 % or fewer study patients, as this would produce a 95 % confidence interval which does not reach 80 %.

We anticipate that routine use of n-of-1 trials might be considered for use in clinical practice if there is no drug that is preferred for ≥ 80 % of patients needing treatment. If no drug is preferred for a high proportion of patients, we will also conduct exploratory analyses to predict which drug is most likely to be preferred for individual patients based on their baseline characteristics (race/ethnicity, gender, age, physical activity). These analyses will be conducted using hierarchical logistic models including the baseline covariates as predictors and subject-specific random effects (to account for within-subject correlation).

We will also employ a Bayesian approach to analysis. Prior probabilities that any drug will be selected as a preferred drug are completely uninformed, given the lack of previous head-to-head comparisons of antihypertensive drugs in children. Each drug will be assigned a prior probability of 33 % (with 95 % credible intervals 10–90 %) to be selected as a preferred drug. We will update the overall probabilities after all n-of-1 trials are complete, and report point estimate and credible intervals for the primary outcome. We will calculate the probability that this proportion is > 80 %. Assuming a prior probability of 33 % and a sample size of 50, if the true rate of drug A being a preferred drug is 60 %, we will have ≥ 90 % power to reject our primary hypothesis.

In short, we will have sufficient sample size to determine if a single drug is preferred by the overwhelming majority of patients.

### Data management

Study data is managed using REDCap (Research Electronic Data Capture) hosted at UTHealth [52]. REDCap is a secure, web-based application designed to support data capture for multiple purposes, including research and quality improvement.

### Ethics approval

The Committee for the Protection of Human Subjects at the UTHealth McGovern Medical School assessed the study protocol and determined that formal approval was not required, as they considered the proposal likely to reduce patient risk and classified this study as a quality improvement project (HSC-MS-13-0287). Verbal informed consent is obtained from all participants. Patients are made aware that their participation is voluntary, and they can withdraw at any time. The question of whether n-of-1 trials should be considered clinical care, quality improvement, and/or research has been discussed elsewhere [53]. Traditional distinctions between patient care and quality improvement initiatives have been seriously questioned with a growing belief that the important issue is whether patient risk is increased relative to usual patient care [54, 55]. Since each therapy tested in the n-of-1 trials is commonly used in routine clinical practice for the treatment of hypertension in children, we do not expect any additional risk to the participants relative to usual patient care. This series of n-of-1 trials is designed to optimize individual patient care in our center, although we expect some generalizable insights to result from our data.

## Discussion

As more children are prescribed antihypertensive therapy for primary hypertension, the need for comparative effectiveness data on the therapeutic options has become more pressing. N-of-1 trials provide physicians with this data from their own patients, allowing the objective evaluation of potentially beneficial therapies while minimizing bias, reducing the time spent on therapies which do not work for that patient, and resolving therapeutic uncertainty much faster than usual care.

### Trial status

The study opened to recruitment in June 2013, with recruitment expected to end in December 2015. The future reports from this study will follow the CONSORT extension for reporting n-of-1 trials (CENT) [56].

## Abbreviations

ABPM: ambulatory blood pressure monitoring
ACE: angiotensin-converting enzyme
BP: blood pressure
DBP: diastolic blood pressure
HPAHP: Houston Pediatric and Adolescent Hypertension Program
RCT: randomized clinical trial
SBP: systolic blood pressure
